# Quality Assessment of Commercial Spagyric Tinctures of *Harpagophytum procumbens* and Their Antioxidant Properties

**DOI:** 10.3390/molecules24122251

**Published:** 2019-06-17

**Authors:** Pinarosa Avato, Maria Pia Argentieri

**Affiliations:** Dipartimento di Farmacia-Scienze del Farmaco, Università degli Studi di Bari Aldo Moro, via Orabona 4, 70125 Bari, Italy; mariapia.argentieri@uniba.it

**Keywords:** *Harpagophytum procumbens*, devil’s claw, harpagoside, spagyric tincture, antioxidant activity

## Abstract

Preparations from the dried tubers of *Harpagophytum procumbens* (Burch.) DC ex Meisn, commonly known as devil’s claw, are mainly used in modern medicine to relieve joint pain and inflammation in patients suffering from rheumatic and arthritic disorders. This paper describes for the first time the chemical profile of a commercial spagyric tincture (named 019) prepared from the roots of the plant. For comparison purposes, a commercial not-spagyric devil’s claw tincture (NST) was also analyzed. Chemical investigation of the content of specialized metabolites in the three samples indicated that harpagoside was the main compound, followed by the two isomers acteoside and isoacteoside. Compositional consistence over time was obtained by the chemical fingerprinting of another spagyric tincture (named 014) from the same producer that was already expired according to the recommendation on the label of the product. The two spagyric preparations did not show significant compositional differences as revealed by HPLC and MS analyses, except for a decrease in harpagide content in the expired 014 tincture. Moreover, their antioxidant capacities as assessed by 2,2’-di-phenyl-1-picrylhydrazyl (DPPH) and 2.2’-azin-bis (3-ethylbenzothiazoline-6-sulfonic acid) (ABTS) methods resulted in very similar IC50 values. The expired 014 tincture showed instead a lower IC50 value compared to the 019 and NST tinctures with the ferric reducing antioxidant potential (FRAP) assay, indicating a higher ferric-reducing antioxidant ability. Overall, these results indicated that the two preparations could generally maintain good stability and biological activity at least for the four years from the production to the expiration date.

## 1. Introduction

*Harpagophytum procumbens* (Burch.) DC ex Meisn, commonly known as devil’s claw, is a plant native to southern Africa, where it is traditionally used as a bitter tonic and a stomachic and also for the treatment of fever, gout, myalgia, and arthritis [1,2]. Accordingly, preparations from the dried tubers of the plant are mainly used in modern medicine and as health products to relieve joint pain and inflammation in patients suffering from rheumatic and arthritic disorders. Clinical studies have in fact shown that extracts from the roots of this plant are good therapeutic remedies for degenerative diseases of the musculoskeletal and locomotor systems [1,3]. In addition, the use of devil’s claw secondary tubers is also approved in preparations for the relief of mild digestive disorders and for the temporary loss of appetite [4,5].

The European Medicines Agency (EMA) recommends preparations obtained by comminuting or powdering the roots of the plant. Liquid, soft, or dry extracts for oral use made in ethanol or water are also included in the recommendation [4,5]. Spagyric tinctures based on *H. procumbens* are also available on the market for articular and rheumatic complaints.

The manufacturing of a spagyric tincture is based on two main stages, namely maceration and fermentation of the drug in alcohol. Processing of the plant material is accomplished in dark thick-walled glass flasks hermetically closed and maintained in greenhouses under a controlled temperature of about 37 °C for 28 days. After this period, the tincture is decanted, and the drug residue is removed from the solution, completely dried, and burnt to ashes to recover the inorganic elements of the plant material. Ashes from the calcined drug are then dissolved in the alcoholic solution from maceration, and the final spagyric preparation is left to stand for 12 days before use.

The practice of “spagyria” dates back to Paracelsus (1493–1541), who first coined the term to mean “separate” (*spao*) and “combine” (*ageiro*), indicating that spagyric preparations are based on “separation”, “extraction”, and “recombination” of the active extractives, including mineral components [6].

The quality control of herbal products aims to assure their consistency, efficacy, and safety. The overall quality of botanicals depends on several factors, including the selection of raw plant materials, harvest time, seasonal changes, and post-harvest processing, and it also relies on extraction procedures and product preparations. All of these factors can primarily affect the plant/drug content of bioactive constituents and impair the efficacy of the final formulation.

The determination of chemical fingerprinting can be a powerful tool to verify the quality of botanicals [7,8,9,10]. Thus, the selection of specific chemical markers of a drug sample/preparation is considered to be crucial for the analysis of its quality. According to the EMA, chemical markers are of interest for quality control regardless of whether they possess any therapeutic or physiological activity, although ideal chemical markers should be unique metabolites that contribute to the biological efficacy of the drug or its preparation [11,12].

The use of fingerprinting in the analytical control of herbal preparation is now a widely accepted official method [7,8,9,10] to assess quality and stability over time and eventually disclose some variation in the chemical content that could reflect a decline in biological activity.

Bitter iridoid glucosides such as harpagoside and harpagide are widely regarded as the active constituents responsible for the pharmacological anti-inflammatory properties of *H. procumbens* [13,14,15,16]. Other components reported as present in the drug are the iridoid procumbide, 8-*O*-*p*-coumaroyl-harpagide, 6′-*O*-*p*-coumaroyl-procumbide, phenylethyl glycosides (such as acteoside and isoacteoside), polyphenolic acids, and flavonoids. All of these phytochemicals are considered to have antioxidant properties and to provide protection against lipid peroxidation [17]. According to the *European Pharmacopoeia* [18], roots from *H. procumbens* must contain ≥1.2% harpagoside. Therefore, a chromatographic profile based on the content of the above main components, taken as the reference chemical markers, should be used in the analytical control of preparations from the roots of *H. procumbens*.

The present manuscript aims to assess quality in terms of the quantitative and qualitative fingerprinting of selected chemical markers of a commercial spagyric preparation based on *H. procumbens*. Compositional data of specialized metabolites were compared to those from a commercial not-spagyric devil’s claw tincture (NST) with June 2020 as the declared expiration date. In addition, an evaluation of possible deterioration of the chemical profile was carried out through chemical fingerprinting of an *H. procumbens* spagyric food supplement from the same manufacturer that was already expired according to the recommendation published on the label of the product. Finally, the antioxidant activity of the two spagyric preparations was determined and compared to possibly reveal any variation in activity due to the aging of the products and degradation of the bioactive phytochemicals.

## 2. Results

### 2.1. Chemical Analysis

**Figure 1:**
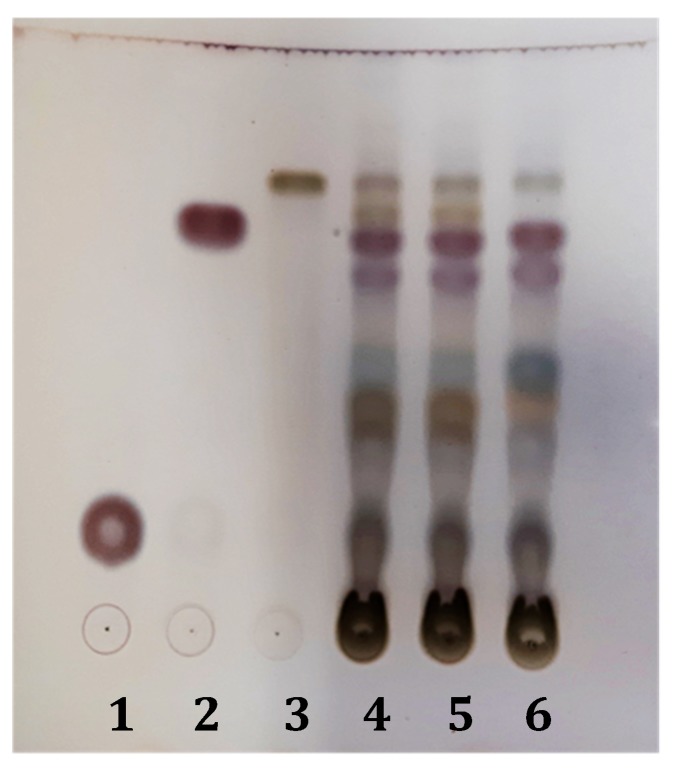
TLC analysis of the two spagyric tinctures, 014 and 019. Mobile phase: EtOAc/HCOOH/CH_3_CO_2_H/H_2_O (100:11:11:26 *v*/*v*); visualization with AS (see Section 4.4). Harpagide, 1; harpagoside, 2; acteoside, 3; 014, 4; 019, 5; not-spagyric tincture (NST), 6.

As shown in Figure 1, the two spagyric tinctures 014 and 019 did not show significant compositional differences as revealed by thin layer chromatography-TLC. No substantial compositional differences were also observed when compared to the NST. As indicated, harpagoside, acteoside, and harpagide could be detected in all of the samples as the main specialized metabolites.

**Figure 2:**
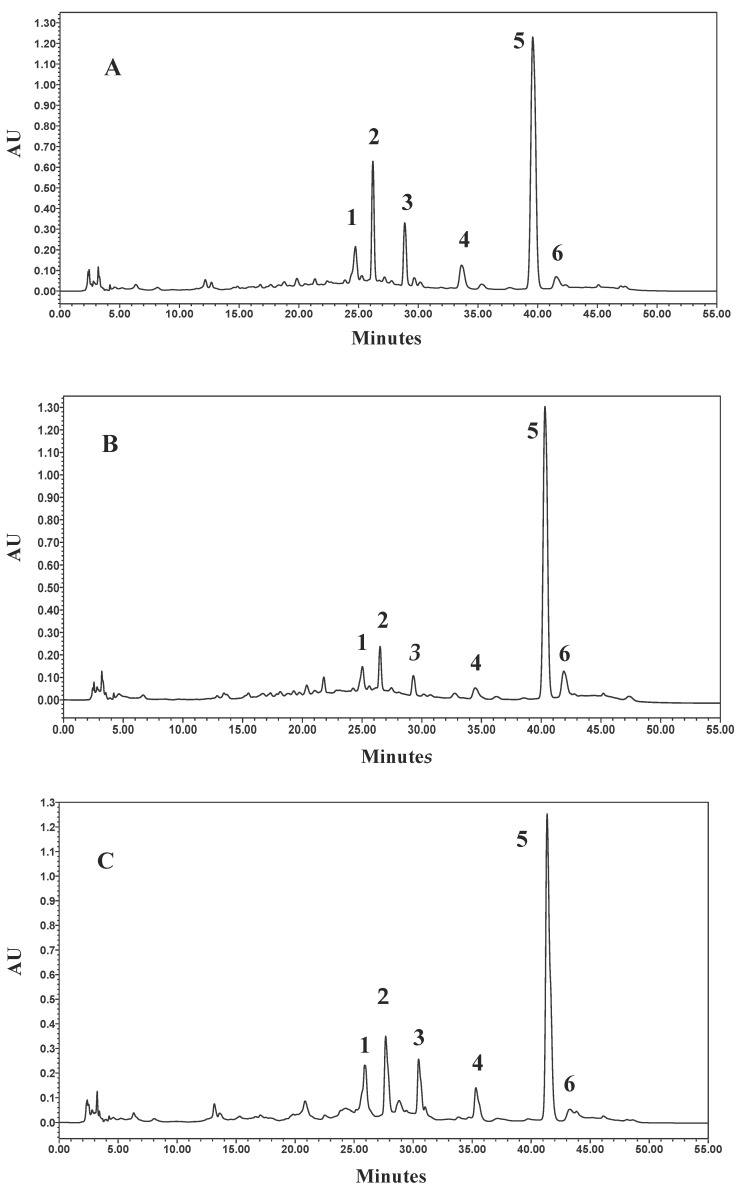
HPLC-DAD (270 nm) chromatograms of the tinctures 019 (**A**), 014 (**B**), and the NST (**C**). For peak naming, see Table 1.

In agreement with TLC analyses, a comparison of the HPLC-DAD (diode-array detector) chromatograms indicated that the three samples 019, 014, and NST did not differ in their general composition (Figure 2). Typical UV absorptions (Table 1) detecting around 217, 246–248, 290, and 325–330 nm for compounds **1** and **2** indicated the presence of phenylethanoid glycosides [19], while the detection of UV maxima at 217 and 280 nm were consistent with an iridoid enol ether system, including a cinnamoyl chromophore in compounds **5** and **6** [20]. In addition, UV absorptions at around 224 and 311 nm suggested the presence of *p*-coumaric acid moieties in compounds **3** and **4**.

Consistent with the above observations, the chemical profile investigated by combining HPLC-DAD and LC–MS/MS and through spiking it with available reference compounds confirmed that the main peak (*t_R_* = 39.58 min) in both samples was represented by harpagoside (Figure 2, peak 5).

**Figure 3:**
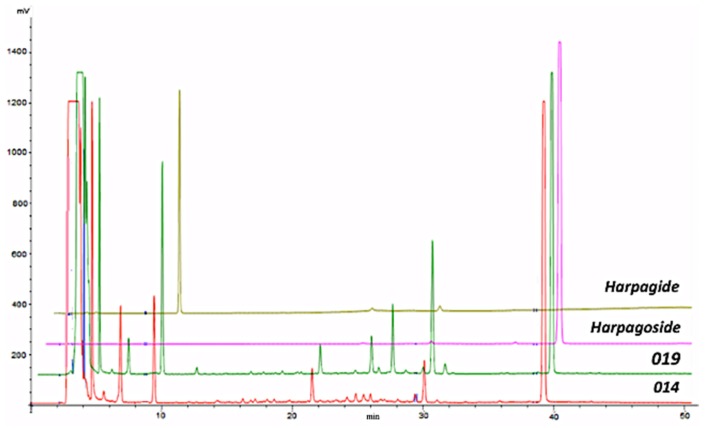
HPLC-ELSD chromatograms of the two spagyric tinctures, 014 and 019: Harpagoside and harpagide.

The UV spectrum showing two absorption bands at 217.7 and 280.4 (max) nm and the LC–MS/MS fragmentation pattern ([*m*/*z* 1011.5 (5) 2M + Na]^+^, [*m*/*z* 517.2 (100) M + Na]^+^, [*m*/*z* 369.2 (100) (M + Na)-148, cinnamic acid]^+^, [*m*/*z* 351.0 (44) (M + Na)-148-18]^+^, [*m*/*z* 203.0 (6) (M + Na)-148-166, harpagide]^+^) were in fact unequivocally consistent with the identification of harpagoside [12,17]. The identity of harpagoside was also acquired by its isolation as a pure constituent through a fast and reliable solid-phase extraction (SPE) optimization method (see Section 4.4). The other major peaks (Figure 2) were respectively identified as peak 1, acteoside (verbascoside), (*t_R_* = 24.70 min), UV 216.6, 246.5 (sh), 290.5 (sh), and 325.7 (max) nm, fragmentation pattern ([*m*/*z* 623.0 (100) M − H]^−^, [*m*/*z* 461 (100) (M − H)-162, glucose]^−^, [*m*/*z* 315.0 (4) (M − H)-162-146, rhamnose]^−^, [*m*/*z* 179.0 (2), caffeoyl moiety]^−^); peak 2, isoacteoside, (*t_R_* = 26.20 min), UV 217.7, 248.5 (sh), 292.3 (sh), and 326.9 (max) nm, fragmentation pattern ([*m*/*z* 623.0 (100) M − H]^−^, [*m*/*z* 461 (100) (M − H)-162, glucose]^−^, [*m*/*z* 315.0 (4) (M − H)-162-146, rhamnose]^−^, [*m*/*z* 179.0 (2), caffeoyl moiety]^−^); peak 3, 8-p-coumaroyl-harpagide, (*t_R_* = 28.87 min), UV 224.8, 311.4 (max) nm, fragmentation pattern ([*m*/*z* 533.1 (100) M + Na]^+^, [*m*/*z* 369.2 (100) (M + Na)-164, coumaric acid]^+^, [*m*/*z* 351.0 (44) (M + Na)-164-18]^+^, [*m*/*z* 203.0 (6) harpagide-162, glucose]^+^); peak 4, pagoside, (*t_R_* = 33.60 min), UV 224.8 and 311.4 (max) nm, fragmentation pattern ([*m*/*z* 491.0 (100) M − H]^−^, [*m*/*z* 311.2 (100) (M − H)-18-162, glucose]^−^, [*m*/*z* 163.0 (31), coumaric acid]^−^); peak 6, 8-cinnamoylmyoporoside (*t_R_* = 41.64 min), UV 217.7 (max) and 278.1 nm, fragmentation pattern ([*m*/*z* 501.0 (100) M + Na]^+^, [*m*/*z* 353.1 (100) (M + Na)-148, cinnamic acid]^+^, [*m*/*z* 335.2 (69) (M + Na)-148-18]^+^, [*m*/*z* 339.0 (41) (M + Na)-162, glucose]^+^, [*m*/*z* 203.0 (6) harpagide-162, glucose]^+^). The presence of harpagide (lacking absorbance at 270 nm), already detected by TLC, was confirmed by HPLC-ELSD (evaporative light scattering detector) analysis (Figure 1 and Figure 3) and by coelution with an authentic compound, and it was in addition identified through its mass spectrum: ([*m*/*z* 365.0 (100) M + H]^+^, [*m*/*z* 347.1 (36) (M + H)-18]^+^, [*m*/*z* 305.1 (75) (M + H)-42-18]^+^, [*m*/*z* 275.1 (41) (M + H)-90]^+^, [*m*/*z* 203.0 (100) (M + H)-162]^+^, [*m*/*z* 185.0 (68) (M + H)-162-18]^+^). The identification of constituents of the two spagyric tinctures was also supported by the available literature [13,21,22,23,24].

In addition to a similar chemical profile, the two spagyric tinctures also showed to a large extent a comparable quantitative composition (Table 2). A quantification of harpagoside as determined by HPLC-DAD against the calibration curve indicated that the compound was equally abundant in the two extracts, amounting respectively to 14.97 ± 0.09 µg/mg ext (59.47%) in the 019 sample and to 13.68 ± 0.19 µg/mg ext (69.68%) in the 014 sample. Other major metabolites were represented by acteoside (019: 1.99 ± 0.23 µg/mg ext, 7.90%; 014: 1.49 ± 0.02 µg/mg ext, 7.59%) and isoacteside (019: 3.94 ± 0.006 µg/mg ext, 15.65%; 014: 1.87 ± 0.14 µg/mg ext, 9.52%). A relatively higher amount of 8-*O*-*p*-coumaroyl-harpagide (019: 2.21 ± 0.0003 µg /mg ext, 8.78%; 014: 0.68 ± 0.006 µg/mg ext, 3.46%) and pagoside (019: 1.37 ± 0.01 µg/mg ext 5.44%; 014: 0.53 ± 0.009 µg/mg ext, 2.70%) was detected in the 019 sample, while 8-cinnamoylmyoporoside was more abundant in 014, 1.38 ± 0.009 µg/mg, 7.03%, versus 0.69 ± 0.008 µg/mg ext, 2.74%, in 019. The same specialized metabolites in the NST sample amounted, respectively, to acteoside, 1.35 ± 0.15 µg/mg ext, 7.39%; isoacteoside, 1.55 ± 0.04 µg/mg ext, 8.48%; 8-*O*-*p*-coumaroyl-harpagide, 0.95 ± 0.04 µg/mg ext, 5.20%; pagoside, 0.72 ± 0.02 µg/mg ext, 3.94%; harpagoside, 13.25 ± 0.07 µg/mg ext, 75.52%; 8-cinnamoylmyoporoside, 0.45 ± 0.005 µg/mg ext, 2.46% (Table 2).

Due to the lack of a chromophore in the molecule, harpagide quantitation was achieved by HPLC-ELSD, which indicated that the content of this phytochemical was slightly higher in the 019 preparation (15.98 ± 0.23 µg/mg ext) compared to the 014 sample (8.50 ± 0.32 µg/mg ext). Similarly to the 019 tincture, harpagide extract accounted for 19.53 ± 0.17 µg/mg ext in the NST.

### 2.2. Antioxidant Activity

**Figure 4:**
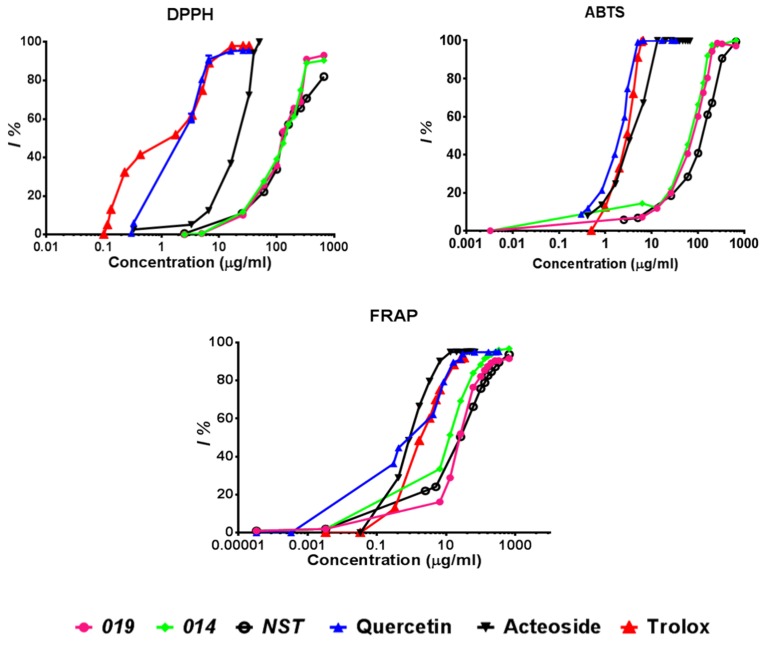
Antioxidant activity of spagyric tinctures 019 and 014, the NST, quercetin, acteoside, and Trolox as a positive control, as determined by DPPH, ABTS, and FRAP methods.

The antioxidant capacity of the two spagyric tinctures of *H. procumbens* was evaluated by in vitro DPPH, ABTS, and FRAP assays (see Section 4.9, Section 4.10 and Section 4.11) and compared to quercetin and acteoside. As shown in Figure 4, the free radical scavenging activity determined by the DPPH and ABTS methods and the ferric-reducing power calculated by the FRAP assay gave almost superimposable behaviors for the two samples, 019 and 014. They displayed a good dose-dependent inhibition of DPPH activity in the range of tested concentrations (up to 89–90% inhibition for 019 and 014, respectively) at 330 μg/mL and up to 98% inhibition of ABTS activity at 260 μg/mL. The ferric-reducing antioxidant power results were instead already high at lower concentrations (52–69% inhibition at 26 μg/mL and up to 76–83% at 60 μg/mL for 019 and 014, respectively).

With regard to the NST sample, its free radical scavenging activity and its ferric-reducing activity (determined by DPPH, ABTS, and FRAP assays) gave results almost superimposable on those obtained for the two spagyric tinctures, with a good dose versus % inhibition correlation.

However, compared to quercetin and acteoside, all three tinctures (019, 014, and the NST) displayed lower antioxidant activity (Figure 4). Both of these two compounds showed antiradical activity reaching 96–100% of DPPH inhibition at concentrations between 26 and 50 μg/mL for quercetin and acteoside. Similarly, quercetin and acteoside were very effective in scavenging the ABTS^+^ radical cation, displaying 100% inhibition between 7 and 13 μg/mL. In addition, they showed discrete FRAP values amounting to 330 (quercetin) and 67 (acteoside) μg/mL to obtain 95% inhibition.

Values of their IC50 (concentration that gives half-maximal response), expressed as mEq of Trolox, are reported in Table 3. Both the 019 and 014 samples showed similar IC50 values of free radical scavenging activity in DPPH and ABTS, amounting to 92.53 ± 0.31 (019) and 93.33 ± 0.25 (014) mEq Trolox and 22.89 ± 0.19 (019) and 19.49 ± 0.13 (014) mEq Trolox, respectively. In contrast to the above, the expired 014 tincture showed a lower IC50 value compared to the 019 tincture in the FRAP method, indicating a higher ferric-reducing antioxidant ability. IC50 values obtained for the NST (Table 3) in the three assays (DPPH, ABTS, and FRAP) were very close to those detected for 019 and even slightly lower, indicating slightly higher antioxidant activity.

The IC50 values of quercetin and acteoside were much lower than those of 019 and 014, indicating the better antioxidant capacity of these two pure reference compounds. Moreover, the IC50 values of quercetin and acteoside, as assessed by the ABTS and FRAP methods, were very similar, while quercetin displayed a more powerful capacity for DPPH reduction than acteoside did (Table 3).

The DPPH assay was also applied to harpagoside, the major iridoid present in the two samples: The IC50 result was higher than 330 ± 0.05 μg/mL, and therefore its antioxidant potency was not tested with the other methods, ABTS and FRAP.

## 3. Discussion

To the best of our knowledge, this is the first report on the phytochemical and biological characterization of spagyric preparations. In this study, we compared the compositional profile of two commercial food supplements (declared to be spagyric tinctures (019 and 014) and provided by the same producer company) with the aim of assessing the quality of the preparations in terms of chemical fingerprinting and biological activity. One of the two spagyric tinctures (014) was already expired according to the recommendation published on the label of the product, and therefore evaluation of the antioxidant activity (using three different methods) was important to display a correlation with the chemical profile and eventually with the aging of the preparation.

The major phytochemicals that have been detected in root extracts from *H. procumbens* include iridoids and phenolic glycosides [13,14,25]. Harpagoside, harpagide, and procumbide are regarded as the most representative iridoid glycosides isolated from the drug, along with the two phenylpropanoid glycosides acteoside and isoacteoside. Several other compounds have been isolated over time in different investigations [13,14,26], such as conjugated esters of iridoid glycosides (8-*O*-*p*-coumaroyl-harpagide, 6′-*O*-*p*-coumaroyl-procumbide, 8-*O*-feruloyl-harpagide, 8-cinnamoylmyoporoside).

As shown in Figure 2, the adopted HPLC analytical method provided a good separation of the phytochemical constituents of the commercial spagyric tinctures. In addition, the method fulfilled all of the validation criteria, showing good linearity of the calibration curve and good limit of detection (LoD) and limit of quantification (LoQ) values, including precision parameters, therefore showing validated repeatability and reproducibility. Compositional requirements in terms of specific chemical markers were also fulfilled in that the two spagyric tinctures, analyzed through TLC, HPLC, UV, and MS methods, displayed the expected phytochemical profile, with harpagoside as the main component along with harpagide, acteoside, and isoacteoside (Table 1).

In addition, the validation of the compositional requirements of both spagyric samples was also confirmed by the analysis of the NST sample, which showed a similar chemical profile, with harpagoside as the most abundant component, followed by acteoside and isoacteoside. Interestingly, the content of harpagide in the NST tincture was comparable (19.53 ± 0.17 µg/mg ext vs 15.98 ± 0.23 µg/mg ext) to that of 019, the tincture that was still not expired.

Spagyric preparations are not conventional herb preparations, but they are based on old alchemic procedures dating back to Paracelsus, who first developed this methodology and described it in his medical treatise *Opus Paramirum*. Our results indicated that the spagyric method allowed us to obtain preparations of good quality based on the presence of marker compounds.

In addition, considering that the two products differed by at least five years in their expiration date and presumably in their production date, fingerprinting analysis and the quantitative determination of the representative constituents in the expired spagyric preparation 014 allowed us to prove a general significant long-term stability of *H. procumbens* active metabolites. A significant decrease of harpagide content was, however, observed in the expired 014 tincture compared to 019 (Table 2).

Preparations based on devil’s claw are mainly used to relieve pain and inflammation due to arthritis and other painful disorders, although the mechanism of action has not yet been fully elucidated. It is known that the generation of oxygen-free radicals is involved in the development of inflammation. Radicals freed from phagocytes are implicated in the activation of nuclear factors, which in turn induces the synthesis of cytokines. Then, a synergic action of oxygen-free radicals and cytokines promotes the synthesis of inflammatory mediators. Maintenance of an adequate antioxidant status may thus help to attenuate the symptoms of inflammation.

There are some indications that the antioxidant properties of some of the specialized metabolites also contribute to the anti-inflammatory effects of pharmaceutical preparations based on *H. procumbens* [14,15,25,27]. However, the antioxidant activity of devil’s claw has not been extensively investigated, and there have been no previous investigations on the antioxidant bioactivity of spagyric preparations. Previous studies have only reported moderate antioxidant activity in methanolic root extracts of *H. procumbens*, while an aqueous extract containing 2.6% harpagoside was shown to have a good capacity to scavenge DPPH. In addition, intraperitoneal administration of a 53% ethanolic extract of devil’s claw to male Wistar rats indicated antioxidant effects similar to those induced by selegiline [4,14,28].

Based on this, we evaluated the antioxidant potency of the two spagyric tinctures to eventually detect any variation in their bioactivity due to the aging of the product and possible degradation of the bioactive phytochemicals. A comparison of the chemicals in and the bioactivity of these two tinctures was also made with a commercial NST.

As for the chemical analysis, significant stability in bioactivity over the long term was also demonstrated by the almost identical antioxidant activity (as determined by the DPPH and ABTS methods (Figure 4)) and the relative IC50 values (Table 3) calculated for the two spagyric preparations, 019 and 014.

An unexpected exception was represented by the lower IC50 value (Table 3) calculated for the expired 014 tincture compared to the 019 tincture in the FRAP method, indicating a higher ferric-reducing antioxidant ability. Reasonably, this might have been due to some species (likely formed upon the aging of the product and not detectable in the used analytical conditions) that were present in the 014 sample and able to efficiently react with the ferric tripyridyltriazine complex.

For instance, it has been reported that the deglycosylation of harpagide gives a degradation product called H-harpagide (2-(formylmethyl)-2,3,5-trihydroxy-5-methylcyclopentane carbaldehyde) that is able to induce the inhibition of the COX-2 enzyme in vitro, in contrast to the parent molecule, which is instead completely inactive [29]. Due to its structure, this molecular species might also undergo oxidation and possibly be more powerful as a ferric-reducing agent. We can suggest that if such a reaction happened upon the aging of the 014 product, it could account for the lower IC50 observed with the FRAP method and the lower amount of harpagide detected in this sample.

Further support for this hypothesis might come from the observation that in the NST tincture, harpagide was present almost in the same amount as in 019 (19.53 ± 0.17 µg/mg ext vs 15.98 ± 0.23 µg/mg ext), and the IC50 values for the antioxidant activity of this sample were very similar to those obtained for the 019 tincture in the three bioassays, including the FRAP test. A deeper investigation should, however, be carried out to clarify this point.

As already reported [13,14,16,30], we also found that acteoside was a powerful antioxidant, while harpagoside did not display antioxidant capacity. Thus, acteoside might reasonably have a role in the antioxidant activity displayed by the two spagyric extracts.

In this study, an evaluation of the antioxidant activity (using three different methods) of the two spagyric tinctures aimed to show a correlation between the chemical profile and eventually the aging of the products. Thus, based on the obtained results, the two analyzed preparations should both be considered to have good biological activity, even when compared to an NST.

## 4. Materials and Methods

### 4.1. Solvents and Reagents

All solvents (HPLC grade and analytical grade) were obtained from Sigma/Aldrich, Italy. Phosphomolybdic acid spray reagent and anisaldehyde for TLC visualization were purchased from Sigma/Aldrich, Italy. Reagents used for the antioxidant bioassays, including DPPH (2,2′-di-phenyl-1-picrylhydrazyl), TPTZ (2,4,6-tripyridyl-*s*-triazine), Trolox (6-hydroxy-2,5,7,8-tetramethylchroman-2-carboxylic acid), and ABTS (2,2′-azino-bis (3-ethylbenzothiazoline-6-sulfonic acid) were all purchased from Sigma/Aldrich, Italy.

### 4.2. Samples

Two commercial samples of food supplements (kindly provided by Erbenobili, Italy) consisting of spagyric tinctures (40% ethanol) of *H. procumbens* roots were used in this study. The two samples differed in their shelf-life date: 2014 and 2019, respectively. That is, one of the two samples, conventionally named 014, was already expired when analyzed, while the other one, named 019, was still valid. For comparison purposes, a commercial NST (40% ethanol) was also analyzed.

### 4.3. Thin Layer Chromatography (TLC)

The composition of devil’s claw extracts 019 and 014 was first investigated by TLC (precoated silica gel 60 F254 aluminum plates, Merck, Milan, Italy) eluted with EtOAc/HCOOH/CH_3_CO_2_H/H_2_O (100:11:11:26 *v*/*v*) or with CHCl_3_/MeOH (90:10 *v*/*v*). Visualization of the components was obtained with phosphomolybdic acid reagent (10% EtOH) or alternatively with anisaldehyde-sulphuric acid reagent (0.5 mL anisaldehyde in 10 mL glacial acetic acid, 85 mL of MeOH in 5 mL of H_2_SO_4_) (AS). After spraying, TLC plates were heated at 110 °C for 5–10 min and observed under visible 254-nm or UV 366-nm light. For TLC and further analyses, the two spagyric tinctures 019 and 014 and the reference NST were died under vacuum and redissolved in 80% MeOH to obtain a 60-mg/mL solution.

### 4.4. Harpagoside Isolation

Harpagoside purification from the two tinctures 019 and 014 was achieved by solid-phase extraction (SPE) on Waters Sep-Pak Vac 1 cc C18 cartridges by using an SPE Extraction Manifold-20 position (Waters, Italy). Cartridges were first conditioned with 5 mL of MeOH and with 10 mL of H_2_O in that order. Then, 1 mL from each of the two samples 019 and 014 was dried under vacuum, redissolved in 80% MeOH, and loaded onto the SPE cartridge. Harpagoside elution was obtained by flushing the cartridge with H_2_O (5 × 1 mL), followed by subsequent elutions with MeOHaq 10% (5 × 1 mL), MeOHaq 50% (5 × 1 mL), and finally MeOHaq 70% (5 × 1 mL). All of the fractions were analyzed by TLC and HPLC. Pure harpagoside was obtained in fractions eluted with MeOHaq 70%.

### 4.5. HPLC-DAD-ELSD

HPLC-DAD-ELSD analyses were performed with a Waters HPLC 600 Liquid Chromatograph equipped with a diode array detector, DAD 2998 Waters, and an evaporative light scattering detector, ELSD 2424 Waters. Data were processed with EmpowerTM 2 Waters Software (Waters S.p:A, Sesto San Giovanni, Milan, Italy). Nitrogen was used as the driving gas for nebulization at 60 psi. Helium was used as the degassing solvent. Extracts were separated with a Gemini C18 (Phenomenex) column (250 × 4.60 mm, 5-µm particle size) equipped with a Security Guard C18 Cartridge (4 × 3 mm, Phenomenex). The following elution system was used with DAD detection: Solvent A, H_2_O; solvent B, CH_3_CN. The elution gradient was 5% B in A increasing to reach 25% B at 20 min and 40% B at 40 min. UV spectra of each extract were conventionally recorded at 210, 270, 310, and 350 nm. The presence of harpagide in the two samples was monitored by acquiring the HPLC-DAD trace at 210 nm. Alternatively, the presence of harpagide was detected by ELSD at the following parameters: Drift tube temperature, 55 °C; gain, 1000. Nitrogen was used as the driving gas for nebulization at 60 psi. Helium was used as the degassing solvent. Reference harpagoside and harpagide were analyzed in the same analytical conditions. All of the HPLC analyses were run in triplicate.

### 4.6. Validation

The quantification of iridoid components in the tinctures under investigation was made by HPLC-DAD against a calibration curve obtained with harpagoside (Phytolab) at six different concentrations in the linear range of 1000–31 µg/mL in MeOHaq 80%. The correlation coefficient (*r^2^*) of the standard curve in the linear plot was *r^2^* = 0.9999 (*y* = 4*E* + 07*x* + 391,135), indicating good linearity between peak areas and concentrations within the used concentration range. The quantitation of harpagide was made by HPLC-ELSD against a calibration curve obtained with harpagide at six different concentrations in the linear range of 2000–62.5 µg/mL. The correlation coefficient (*r*^2^) of the standard curve in the linear plot of log-transformed data was *r*^2^ = 0.9992 (*y* = 1.7569*x* − 1.7101), indicating good linearity between the log-transformed areas and log-transformed concentrations within the tested concentration range.

The limit of detection (LoD) and limit of quantification (LoQ) were calculated according to conventional guidelines [31,32] on the basis of signal-to-noise ratios (S/Ns) of 3:1 and 10:1, respectively, by injecting a series of dilute solutions of known concentrations of harpagoside in the range of 31–0.061 μg/mL. The LoD for harpagoside in the adopted analytical conditions was 0.061 µg/mL, while the LoQ was 0.210 µg/mL.

The precision of the analytical HPLC method was assessed by calculating intraday repeatability and interday intermediate precision [33]. Multiple injections of harpagoside solutions (0.03125 µg/mL) were performed within the same day for intraday precision, while for interday precision, analyses were run in triplicate over three consecutive days. Precision was expressed as the percent relative standard deviation (RSD%) of retention times and peak areas resulting from the analysis of the spagyric tinctures. The intraday and interday variabilities of harpagoside in the two spagyric tinctures 019 and 014 were also calculated with the same procedure. Intraday precision of the chromatographic method for harpagoside was observed in the range of 0.25–0.59% for retention times and between 0.87% and 0.90% for peak areas. Interday RSD values were 0.28% and 0.95% for retention times and peak areas, respectively. The intraday RSD values of retention times and peak areas for the 6 major analytes in the spagyric tinctures were in the range of 0.10–1.10% and 0.50–3.00%, respectively. Percent RSD values of retention times and peak areas for interday evaluation were in the range of 0.21–3.47% and 0.17–3.55%, respectively. Overall, the results obtained indicated that the method applied for the compositional analysis was precise and accurate.

### 4.7. LC-ESI-MS/MS

Flow injection LC-ESI-MS/MS analyses were performed on a 1100 Series Agilent LC/MSD Trap-System VL (Agilent Technologies Italia SpA, Cernusco sul Naviglio, Milan, Italy). The chromatographic conditions were as described above for the HPLC-DAD analyses. An Agilent Chemstation (LC/MSD TrapSoftware 4.1, (Agilent Technologies Italia SpA, Cernusco sul Naviglio, Milan, Italy) was used for the acquisition and processing of the data. The mass spectrometer operated in the positive and negative ion mode under the following settings: Capillary voltage, 47 V; nebulizer gas (N_2_), 5 psi; drying gas (N_2_), heated at 220 °C and introduced at a flow rate of 10 µL/mL. Data were acquired in the MS scanning mode over the range of 150–1500 *m*/*z* with a scan time of 13000 *m*/*z*/s. Automated MS/MS was performed by isolating the base peaks (pseudomolecular ions) using an isolation width of 4.0 *m*/*z*, a fragmentation amplitude of 1.0 V, the threshold set at 100, and the ion charge control on, with the maximum acquire time set at 300 ms. Samples were dissolved in MeOHaq 80% and injected at a flow rate of 0.2 mL/min.

### 4.8. Antioxidant Activity

Antioxidant activity of the three tinctures of *H. procumbens* (019, 014, and NST) was determined by different methods: DPPH, ferric-reducing antioxidant power (FRAP) assay, and Trolox/ABTS equivalent antioxidant capacity [34,35,36]. For comparative purposes, the antioxidant activity of quercetin (Sigma/Aldrich, Italy), harpagoside (Phytolab, Germany), and acteoside (Sigma-Aldrich S.r.l., Milan, Italy) were also determined with the same procedures. Determinations were carried out in triplicate.

### 4.9. DPPH

The scavenging activity for DPPH (Sigma, Milan, Italy) free radicals was measured according to Brand–Williams (1995), with some modifications. To 2.9 mL of a 10 × 10^−5^-M solution of DPPH in MeOH, 0.1 mL of a properly diluted MeOH sample test solution was added and vortexed, that is, different dilutions of each test sample (019, 014) were prepared to determine the actual IC50 values. After 30 min of incubation in the dark at room temperature, the UV absorbance (Perkin-Elmer Lambda Bio 20 Spectrophotometer) of each sample was recorded at 515 nm against the blank (reagent solution without the test sample). All determinations were carried out in triplicate. The inhibition percentage (I%) of DPPH radicals in the test samples was calculated with the following equation: *I*% = [(*A*_blank_ − *A*_sample_)/*A*_blank_] x 100, where *A*_blank_ is the absorbance of the blank solution at *t* = 0, and *A*_sample_ is the absorbance of the test sample at *t* = 30 min. The IC50 concentration is the amount of drug necessary to give 50% inhibition of the DPPH radicals. Acteoside, quercetin, and harpagoside were used as reference compounds.

### 4.10. FRAP

The following stock solutions were prepared for the bioassay according to Benzie and Strain (1996): a) a 300-mM acetate buffer solution, pH 3.6; b) a 10-mM TPTZ (2,4,6-tripyridyl-*s*-triazine) solution in MeOH; and c) a 20-mM FeCl_3_·6 H_2_O solution. The FRAP working solution was prepared by mixing TPTZ and FeCl_3_·6 H_2_O and acetate buffer in a ratio of 1:1:10. Test samples and reference compounds (100 μL) were added to 2.6 mL of FRAP reagent, along with 300 μL of H_2_O, and allowed to react for 30 min at 37 °C in dark conditions. Absorbance readings (ferrous tripyridyltriazine complex) were taken at 595 nm. A blank solution consisted of FRAP solutions above and in water. The standard curve was (*r^2^* = 0.9992; *y* = 0.0005*x* + 0.0005) using a concentration range of FeSO_4_ between 0.42 µg/mL and 20 µg/mL.

### 4.11. Trolox/ABTS

Radical scavenging capacity was also studied based on the reduction of ABTS+· radicals by the test samples. An ABTS+· radical cation was obtained by mixing an ABTS stock solution (7 mM in H_2_O) with 2.5 mM of potassium persulfate, K_2_S_2_O_8_. This solution was allowed to stand at room temperature in the dark for 12–16 h before use. For the analyses, the ABTS+· solution was diluted with water to an absorbance of 0.7 (±0.02) at 734 nm. For the UV determinations, 2.9 mL of the ABTS+· solution and 100 μL of each of the test samples were mixed, and an absorbance reading was taken at 734 nm, 2–6 min after mixing. Appropriate ABTS blanks were run in each assay. All solutions were freshly prepared for the daily experiments. The scavenging activity of each test sample was compared to that of Trolox (6-hydroxy-2,5,7,8-tetramethylchroman-2-carboxylic acid) as the positive control. A linear calibration curve (*r*^2^ = 0.9970; *y* = 0.0005*x* + 0.0005) was constructed using Trolox in a range from 4 μM to 26 μM.

### 4.12. Statistical Analysis

The quantitative results for each analysis and bioassay were expressed as the mean ± standard deviation (SD) of at least three replicates for each experiment. Microsoft Office Excel 2017 was used for processing the data.

## Figures and Tables

**Table 1:** MS fragmentation and UV-VIS absorption data of compounds detected in the tinctures of Harpagophytum procumbens by HPLC-DAD (270 nm).

Number *	R_t_ (min)	Name	UV (λ max, nm)	ms1	ms2
				*m*/*z* (%)	
**1**	24.70	Acteoside	216.6, 246.5 sh, 290.5 sh, 325.7	623.0 (100) [M − H]^−^	461.0 (100) [(M − H)-162]^−^; 315.0 (4) [(M − H)-162-146]^−^; 179.0 (2), [caffeoyl moiety] ^−^
**2**	26.20	Isoacteoside	217.7, 248.5 sh, 292.3 sh, 326.9	623.0 (100) [ M − H ]^−^	461.0 (100) [(M-H)-162]^−^; 315.0 (4) [(M − H)-162-146]^−^; 179.0 (2), [caffeoyl moiety]^−^
**3**	28.87	8-*O*-*p*-Coumaroyl-harpagide	224.8, 311.4	533.1 (100) [M + Na]^+^	369.2 (100) [(M + Na)-164]^+^; 351.0 (44) [(M + Na)-164-18]^+^; 203.0 (6) [harpagide-162]^+^
**4**	33.60	Pagoside	224.8, 311.4	491.0 (100) [ M − H ]^−^	311.2 (100) [(M − H)-162-18]^−^; 163.0 (31) [coumaric acid]^−^
**5**	39.58	Harpagoside	217.7, 280.4	1011.5 (5) [2M + Na]^+^ /517.2 (100) [M + Na]^+^	369.2 (100) [(M + Na)-148]^+^; 351.0 (44) [(M + Na)-148-18]^+^; 203.0 (6) [(M + Na)-148-166]^+^
**6**	41.64	8-Cinnamoylmyoporoside	217.7, 278.1	501.0 (100) [M + Na]^+^	353.1 (100) [(M + Na)-148]^+^; 335.2 (69) [(M + Na)-148-18]^+^; 339.0 (41) [(M + Na)-162]^+^; 203.0 (6) harpagide-162]^+^

* For peak numbers, see text and Figure 2.

**Table 2:** Specialized metabolites identified in 019, 014, and the NST.

Compound	014		019		NST	
	μg/mg ext	%	μg/mg ext	%	μg/mg ext	%
1	1.49 ± 0.02	7.59	1.99 ± 0.23	7.90	1.35 ± 0.15	7.39
2	1.87 ± 0.14	9.52	3.94 ± 0.006	15.65	1.55 ± 0.04	8.48
3	0.68 ± 0.006	3.46	2.21 ± 0.0003	8.78	0.95 ± 0.04	5.2
4	0.53 ± 0.009	2.70	1.37 ± 0.01	5.44	0.72 ± 0.02	3.94
5	13.68 ± 0.19	69.68	14.97 ± 0.09	59.47	13.25 ± 0.07	72.52
6	1.38 ± 0.009	7.03	0.69 ± 0.008	2.74	0.45 ± 0.005	2.46

For peak naming, see Table 1.

**Table 3:** Antioxidant activity (IC50) of the spagyric tinctures 019 and 014 and the NST of *H. procumbens*.

Sample	IC50 (mEq Trolox) ± SD		
	DPPH	ABTS	FRAP
019	92.53 ± 0.31	22.89 ± 0.19	14.11 ± 0.08
014	93.33 ± 0.25	19.49 ± 0.13	6.69 ± 0.07
NST	83.02 ± 0.28	17.78 ± 0.12	11.32 ± 0.08
Acteoside	13.83 ± 0.09	0.96 ± 0.05	0.44 ± 0.04
Quercetin	1.38 ± 0.01	0.60 ± 0.07	0.38 ± 0.02
